# Supplementary material for: Alternative splicing across the tree of life
Source: eLife. 2025 Oct 17;13:RP94802. doi: 10.7554/eLife.94802 (PMC12534046; doi:10.7554/eLife.94802)
Supplement: Supplementary file 1. [file elife-94802-supp1.docx]

Pairwise comparisons of ASR and ASR* across taxonomic groups, showing differences in statistical measures (mean and median). Statistical significance was assessed using Monte Carlo permutation tests, and adjusted p-values were calculated using the Bonferroni correction (see Methods). Colors represent significance levels: red for p ≤ 0.001 (***), green for 0.001 < p ≤ 0.05 (**), and blue for 0.05 < p ≤ 0.1 (*). Values with p > 0.1 have no color.

**Differences in Mean Alternative Splicing Ratio (ASR)**

|  | **Mammals** | **Birds** | **Fish** | **Arthropods** | **Plants** | **Fungi** | **Uni. Euk.** | **Bacteria** | **Archaea** |
| --- | --- | --- | --- | --- | --- | --- | --- | --- | --- |
| **Mammals** | 0 | 0.38 * | 0.83 *** | 0.93 *** | 1.53 *** | 2.08 *** | 2.09 *** | 2.09 *** | 2.09 *** |
| **Birds** |  | 0 | 0.45 *** | 0.56 *** | 1.15 *** | 1.70 *** | 1.71 *** | 1.71 *** | 1.71 *** |
| **Fish** |  |  | 0 | 0.11 | 0.70 *** | 1.25 *** | 1.26 *** | 1.26 *** | 1.26 *** |
| **Arthropods** |  |  |  | 0 | 0.60 *** | 1.14 *** | 1.16 *** | 1.15 *** | 1.15 *** |
| **Plants** |  |  |  |  | 0 | 0.55 *** | 0.56 *** | 0.56 *** | 0.56 *** |
| **Fungi** |  |  |  |  |  | 0 | 0.01 | 0.01 *** | 0.01 *** |
| **Uni. Euk.** |  |  |  |  |  |  | 0 | 0.00 | 0.00 *** |
| **Bacteria** |  |  |  |  |  |  |  | 0 | 0.00 *** |
| **Archaea** |  |  |  |  |  |  |  |  | 0 |

**Differences in Median Alternative Splicing Ratio (ASR)**

|  | **Mammals** | **Birds** | **Fish** | **Arthropods** | **Plants** | **Fungi** | **Uni. Euk.** | **Bacteria** | **Archaea** |
| --- | --- | --- | --- | --- | --- | --- | --- | --- | --- |
| **Mammals** | 0 | 0.27 | 0.75 *** | 0.92 *** | 1.47 *** | 2.02 *** | 2.02 *** | 2.02 *** | 2.02 *** |
| **Birds** |  | 0 | 0.49 *** | 0.65 *** | 1.20 *** | 1.75 *** | 1.75 *** | 1.75 *** | 1.75 *** |
| **Fish** |  |  | 0 | 0.17 | 0.72 *** | 1.27 *** | 1.27 *** | 1.27 *** | 1.27 *** |
| **Arthropods** |  |  |  | 0 | 0.55 *** | 1.10 *** | 1.10 *** | 1.10 *** | 1.10 *** |
| **Plants** |  |  |  |  | 0 | 0.55 *** | 0.55 *** | 0.55 *** | 0.55 *** |
| **Fungi** |  |  |  |  |  | 0 | 0.00 | 0.00 *** | 0.00 *** |
| **Uni. Euk.** |  |  |  |  |  |  | 0 | 0.00 *** | 0.00 * |
| **Bacteria** |  |  |  |  |  |  |  | 0 | 0.00 *** |
| **Archaea** |  |  |  |  |  |  |  |  | 0 |

**Differences in Mean Normalized Alternative Splicing Ratio (ASR*)**

|  | **Mammals** | **Birds** | **Fish** | **Arthropods** | **Plants** | **Fungi** | **Uni. Euk.** | **Bacteria** | **Archaea** |
| --- | --- | --- | --- | --- | --- | --- | --- | --- | --- |
| **Mammals** | 0 | 0.33 *** | 0.78 *** | 0.51 *** | 0.70 *** | 1.79 *** | 1.81 *** | 1.81 *** | 1.80 *** |
| **Birds** |  | 0 | 0.46 *** | 0.18 ** | 0.37 *** | 1.46 *** | 1.48 *** | 1.48 *** | 1.48 *** |
| **Fish** |  |  | 0 | 0.27 *** | 0.08 | 1.01 *** | 1.02 *** | 1.02 *** | 1.02 *** |
| **Arthropods** |  |  |  | 0 | 0.19 *** | 1.28 *** | 1.29 *** | 1.29 *** | 1.29 *** |
| **Plants** |  |  |  |  | 0 | 1.09 *** | 1.10 *** | 1.10 *** | 1.10 *** |
| **Fungi** |  |  |  |  |  | 0 | 0.01 | 0.01 *** | 0.01 *** |
| **Uni. Euk.** |  |  |  |  |  |  | 0 | 0.00 | 0.00 *** |
| **Bacteria** |  |  |  |  |  |  |  | 0 | 0.00 *** |
| **Archaea** |  |  |  |  |  |  |  |  | 0 |

**Differences in Median Normalized Alternative Splicing Ratio (ASR*)**

|  | **Mammals** | **Birds** | **Fish** | **Arthropods** | **Plants** | **Fungi** | **Uni. Euk.** | **Bacteria** | **Archaea** |
| --- | --- | --- | --- | --- | --- | --- | --- | --- | --- |
| **Mammals** | 0 | 0.25 *** | 0.69 *** | 0.42 *** | 0.60 *** | 1.74 *** | 1.74 *** | 1.73 *** | 1.73 *** |
| **Birds** |  | 0 | 0.44 *** | 0.17 | 0.35 *** | 1.48 *** | 1.48 *** | 1.48 *** | 1.48 *** |
| **Fish** |  |  | 0 | 0.26 *** | 0.09 | 1.05 *** | 1.05 *** | 1.05 *** | 1.05 *** |
| **Arthropods** |  |  |  | 0 | 0.17 *** | 1.31 *** | 1.31 *** | 1.31 *** | 1.31 *** |
| **Plants** |  |  |  |  | 0 | 1.14 *** | 1.14 *** | 1.14 *** | 1.14 *** |
| **Fungi** |  |  |  |  |  | 0 | 0.00 | 0.00 *** | 0.00 *** |
| **Uni. Euk.** |  |  |  |  |  |  | 0 | 0.00 *** | 0.00 * |
| **Bacteria** |  |  |  |  |  |  |  | 0 | 0.00 *** |
| **Archaea** |  |  |  |  |  |  |  |  | 0 |
