# Supplementary material for: Alternative splicing across the tree of life
Source: eLife. 2025 Oct 17;13:RP94802. doi: 10.7554/eLife.94802 (PMC12534046; doi:10.7554/eLife.94802)
Supplement: Supplementary file 2. [file elife-94802-supp2.docx]

Pairwise comparisons of genomic variables across taxonomic groups, showing differences in statistical measures (mean and median) for gene content relative to genome size, coding size relative to gene content, and coding relative to genome size. Statistical significance was assessed using Monte Carlo permutation tests, and adjusted p-values were calculated using the Bonferroni correction (see Methods). Colors represent significance levels: red for p ≤ 0.001 (***), green for 0.001 < p ≤ 0.05 (**), and blue for 0.05 < p ≤ 0.1 (*). Values with p > 0.1 have no color.

**Differences in Mean Gene Content / Genome Size Ratio**

|  | **Mammals** | **Birds** | **Fish** | **Arthropods** | **Plants** | **Fungi** | **Uni. Euk.** | **Bacteria** | **Archaea** |
| --- | --- | --- | --- | --- | --- | --- | --- | --- | --- |
| **Mammals** | 0 | 0.08 *** | 0.17 *** | 0.16 *** | 0.20 *** | 0.19 *** | 0.16 *** | 0.41 *** | 0.41 *** |
| **Birds** |  | 0 | 0.09 *** | 0.08 *** | 0.28 *** | 0.11 *** | 0.08 ** | 0.33 *** | 0.34 *** |
| **Fish** |  |  | 0 | 0.02 | 0.37 *** | 0.01 | 0.01 | 0.24 *** | 0.24 *** |
| **Arthropods** |  |  |  | 0 | 0.36 *** | 0.03 | 0.01 | 0.25 *** | 0.26 *** |
| **Plants** |  |  |  |  | 0 | 0.39 *** | 0.36 *** | 0.61 *** | 0.61 *** |
| **Fungi** |  |  |  |  |  | 0 | 0.02 | 0.23 *** | 0.23 *** |
| **Uni. Euk.** |  |  |  |  |  |  | 0 | 0.25 *** | 0.25 *** |
| **Bacteria** |  |  |  |  |  |  |  | 0 | 0.00 |
| **Archaea** |  |  |  |  |  |  |  |  | 0 |

**Differences in Median Gene Content / Genome Size Ratio**

|  | **Mammals** | **Birds** | **Fish** | **Arthropods** | **Plants** | **Fungi** | **Uni. Euk.** | **Bacteria** | **Archaea** |
| --- | --- | --- | --- | --- | --- | --- | --- | --- | --- |
| **Mammals** | 0 | 0.08 *** | 0.18 *** | 0.17 *** | 0.19 *** | 0.19 *** | 0.16 *** | 0.42 *** | 0.42 *** |
| **Birds** |  | 0 | 0.10 *** | 0.09 *** | 0.27 *** | 0.11 *** | 0.08 ** | 0.34 *** | 0.34 *** |
| **Fish** |  |  | 0 | 0.01 | 0.36 *** | 0.01 | 0.02 | 0.24 *** | 0.25 *** |
| **Arthropods** |  |  |  | 0 | 0.36 *** | 0.02 | 0.01 | 0.25 *** | 0.25 *** |
| **Plants** |  |  |  |  | 0 | 0.38 *** | 0.34 *** | 0.61 *** | 0.61 *** |
| **Fungi** |  |  |  |  |  | 0 | 0.03 | 0.23 *** | 0.23 *** |
| **Uni. Euk.** |  |  |  |  |  |  | 0 | 0.26 *** | 0.26 *** |
| **Bacteria** |  |  |  |  |  |  |  | 0 | 0.00 |
| **Archaea** |  |  |  |  |  |  |  |  | 0 |

**Differences in Mean Coding Size / Gene Content Ratio**

|  | **Mammals** | **Birds** | **Fish** | **Arthropods** | **Plants** | **Fungi** | **Uni. Euk.** | **Bacteria** | **Archaea** |
| --- | --- | --- | --- | --- | --- | --- | --- | --- | --- |
| **Mammals** | 0 | 0.02 *** | 0.06 *** | 0.08 *** | 0.24 *** | 0.84 *** | 0.86 *** | 0.96 *** | 0.96 *** |
| **Birds** |  | 0 | 0.04 *** | 0.06 *** | 0.23 *** | 0.82 *** | 0.84 *** | 0.94 *** | 0.94 *** |
| **Fish** |  |  | 0 | 0.03 ** | 0.19 *** | 0.78 *** | 0.80 *** | 0.90 *** | 0.90 *** |
| **Arthropods** |  |  |  | 0 | 0.16 *** | 0.76 *** | 0.77 *** | 0.88 *** | 0.88 *** |
| **Plants** |  |  |  |  | 0 | 0.60 *** | 0.61 *** | 0.71 *** | 0.72 *** |
| **Fungi** |  |  |  |  |  | 0 | 0.02 | 0.12 *** | 0.12 *** |
| **Uni. Euk.** |  |  |  |  |  |  | 0 | 0.10 *** | 0.10 *** |
| **Bacteria** |  |  |  |  |  |  |  | 0 | 0.00 *** |
| **Archaea** |  |  |  |  |  |  |  |  | 0 |

**Differences in Median Coding Size / Gene Content Ratio**

|  | **Mammals** | **Birds** | **Fish** | **Arthropods** | **Plants** | **Fungi** | **Uni. Euk.** | **Bacteria** | **Archaea** |
| --- | --- | --- | --- | --- | --- | --- | --- | --- | --- |
| **Mammals** | 0 | 0.02 *** | 0.05 *** | 0.07 *** | 0.25 *** | 0.87 *** | 0.87 *** | 0.96 *** | 0.96 *** |
| **Birds** |  | 0 | 0.04 *** | 0.05 *** | 0.23 *** | 0.85 *** | 0.85 *** | 0.94 *** | 0.94 *** |
| **Fish** |  |  | 0 | 0.02 ** | 0.20 *** | 0.82 *** | 0.81 *** | 0.90 *** | 0.91 *** |
| **Arthropods** |  |  |  | 0 | 0.18 *** | 0.80 *** | 0.80 *** | 0.89 *** | 0.89 *** |
| **Plants** |  |  |  |  | 0 | 0.62 *** | 0.61 *** | 0.71 *** | 0.71 *** |
| **Fungi** |  |  |  |  |  | 0 | 0.01 | 0.08 *** | 0.09 *** |
| **Uni. Euk.** |  |  |  |  |  |  | 0 | 0.09 *** | 0.10 *** |
| **Bacteria** |  |  |  |  |  |  |  | 0 | 0.00 *** |
| **Archaea** |  |  |  |  |  |  |  |  | 0 |

**Differences in Mean Coding Content / Genome Size Ratio**

|  | **Mammals** | **Birds** | **Fish** | **Arthropods** | **Plants** | **Fungi** | **Uni. Euk.** | **Bacteria** | **Archaea** |
| --- | --- | --- | --- | --- | --- | --- | --- | --- | --- |
| **Mammals** | 0 | 0.01 *** | 0.04 *** | 0.06 *** | 0.05 *** | 0.54 *** | 0.52 *** | 0.83 *** | 0.84 *** |
| **Birds** |  | 0 | 0.03 *** | 0.04 *** | 0.04 *** | 0.53 *** | 0.51 *** | 0.82 *** | 0.83 *** |
| **Fish** |  |  | 0 | 0.02 *** | 0.01 ** | 0.50 *** | 0.48 *** | 0.79 *** | 0.80 *** |
| **Arthropods** |  |  |  | 0 | 0.00 | 0.48 *** | 0.47 *** | 0.78 *** | 0.78 *** |
| **Plants** |  |  |  |  | 0 | 0.49 *** | 0.47 *** | 0.78 *** | 0.78 *** |
| **Fungi** |  |  |  |  |  | 0 | 0.02 | 0.29 *** | 0.30 *** |
| **Uni. Euk.** |  |  |  |  |  |  | 0 | 0.31 *** | 0.32 *** |
| **Bacteria** |  |  |  |  |  |  |  | 0 | 0.01 |
| **Archaea** |  |  |  |  |  |  |  |  | 0 |

**Differences in Median Coding Content / Genome Size Ratio**

|  | **Mammals** | **Birds** | **Fish** | **Arthropods** | **Plants** | **Fungi** | **Uni. Euk.** | **Bacteria** | **Archaea** |
| --- | --- | --- | --- | --- | --- | --- | --- | --- | --- |
| **Mammals** | 0 | 0.01 *** | 0.04 *** | 0.05 *** | 0.05 *** | 0.54 *** | 0.51 *** | 0.84 *** | 0.85 *** |
| **Birds** |  | 0 | 0.03 *** | 0.04 *** | 0.04 *** | 0.52 *** | 0.50 *** | 0.83 *** | 0.83 *** |
| **Fish** |  |  | 0 | 0.01 *** | 0.01 *** | 0.50 *** | 0.47 *** | 0.80 *** | 0.81 *** |
| **Arthropods** |  |  |  | 0 | 0.00 | 0.49 *** | 0.46 *** | 0.79 *** | 0.80 *** |
| **Plants** |  |  |  |  | 0 | 0.49 *** | 0.46 *** | 0.79 *** | 0.80 *** |
| **Fungi** |  |  |  |  |  | 0 | 0.03 | 0.30 *** | 0.31 *** |
| **Uni. Euk.** |  |  |  |  |  |  | 0 | 0.33 *** | 0.34 *** |
| **Bacteria** |  |  |  |  |  |  |  | 0 | 0.01 |
| **Archaea** |  |  |  |  |  |  |  |  | 0 |
