# Supplementary material for: Alternative splicing across the tree of life
Source: eLife. 2025 Oct 17;13:RP94802. doi: 10.7554/eLife.94802 (PMC12534046; doi:10.7554/eLife.94802)
Supplement: Supplementary file 3. [file elife-94802-supp3.docx]

PGLS analysis (Y ~ βX). For each taxonomic group, results are shown for six relationships (ASR and ASR* vs Genome Size, Gene Content, Coding, Gene/Genome, Coding/Gene, and Coding/Genome). We report β, adjusted R² (R²_adj), and Pagel's λ. Significance coloring on β: red p ≤ 0.001 (***), green 0.001 < p ≤ 0.05 (**), blue 0.05 < p ≤ 0.1 (*). Values with p > 0.1 have no color.

**Part A: ASR models**

**Mammals**

| **Relationship** | **β** | **R²_adj** | **λ** |
| --- | --- | --- | --- |
| **vs. Genome Size** | 5.0 x 10^-4 | 0.01 | 0.35 |
| **vs. Gene Content** | 4.0 x 10^-3*** | 0.55 | 0.41 |
| **vs. Coding** | 0.28*** | 0.16 | 0.41 |
| **vs. Gene / Genome** | 9.95*** | 0.43 | 0.49 |
| **vs. Coding / Gene** | -184.05*** | 0.52 | 0.52 |
| **vs. Coding / Genome** | -8.25 | -0.01 | 0.39 |

**Birds**

| **Relationship** | **β** | **R²_adj** | **λ** |
| --- | --- | --- | --- |
| **vs. Genome Size** | 5.0 x 10^-4 | -0.01 | 0.75 |
| **vs. Gene Content** | 7.0 x 10^-3*** | 0.70 | 0.97 |
| **vs. Coding** | 0.23*** | 0.27 | 0.25 |
| **vs. Gene / Genome** | 8.11*** | 0.58 | 0.62 |
| **vs. Coding / Gene** | -152.85*** | 1.00 | 1.00 |
| **vs. Coding / Genome** | 87.09** | 0.05 | 0.36 |

**Fish**

| **Relationship** | **β** | **R²_adj** | **λ** |
| --- | --- | --- | --- |
| **vs. Genome Size** | -2.0 x 10^-4* | 0.02 | 0.59 |
| **vs. Gene Content** | 7.6 x 10^-5 | -4.6 x 10^-3 | 0.50 |
| **vs. Coding** | 5.0 x 10^-3 | 8.0 x 10^-4 | 0.47 |
| **vs. Gene / Genome** | 4.51*** | 0.50 | 0.75 |
| **vs. Coding / Gene** | -1.39 | -8.4 x 10^-4 | 0.6 |
| **vs. Coding / Genome** | 6.34** | 0.04 | 0.61 |

**Arthropods**

| **Relationship** | **β** | **R²_adj** | **λ** |
| --- | --- | --- | --- |
| **vs. Genome Size** | -1.0 x 10^-4 | 0.01 | 0.59 |
| **vs. Gene Content** | -5.3 x 10^-5 | -0.01 | 0.59 |
| **vs. Coding** | -0.01 | 2.5 x 10^3 | 0.59 |
| **vs. Gene / Genome** | 2.24*** | 0.27 | 0.71 |
| **vs. Coding / Gene** | -1.24 | 0.01 | 0.66 |
| **vs. Coding / Genome** | 1.29 | 3.2 x 10^-4 | 0.58 |

**Plants**

| **Relationship** | **β** | **R²_adj** | **λ** |
| --- | --- | --- | --- |
| **vs. Genome Size** | -9.2 x 10^-6 | -0.01 | 0.90 |
| **vs. Gene Content** | 4.8 x 10^-4** | 0.04 | 0.93 |
| **vs. Coding** | -4.5 x 10^-4 | -0.01 | 0.90 |
| **vs. Gene / Genome** | 0.33** | 0.03 | 0.89 |
| **vs. Coding / Gene** | -1.55*** | 0.26 | 0.93 |
| **vs. Coding / Genome** | -0.36 | -4.4 x 10^-3 | 0.91 |

**Fungi**

| **Relationship** | **β** | **R²_adj** | **λ** |
| --- | --- | --- | --- |
| **vs. Genome Size** | 2.0 x 10^-4 | -0.01 | 0.50 |
| **vs. Gene Content** | 3.8 x 10^-3** | 0.11 | 0.50 |
| **vs. Coding** | 1.7 x 10^-3 | -8.5 x 10^-4 | 0.50 |
| **vs. Gene / Genome** | 0.14** | 0.06 | 0.20 |
| **vs. Coding / Gene** | -0.35*** | 0.26 | 0.50 |
| **vs. Coding / Genome** | -0.1 | -0.01 | 0.5 |

**Unicellular Eukaryotes**

| **Relationship** | **β** | **R²_adj** | **λ** |
| --- | --- | --- | --- |
| **vs. Genome Size** | -9.2 x 10^-6 | -0.02 | 0.25 |
| **vs. Gene Content** | -2.0 x 10^-5 | -0.2 | 0.22 |
| **vs. Coding** | -1.2 x 10^-5 | -0.03 | 0.25 |
| **vs. Gene / Genome** | -1.4 x 10^-4 | -0.03 | 0.25 |
| **vs. Coding / Gene** | 0.01 | -4.6 x 10^-3 | 0.25 |
| **vs. Coding / Genome** | 3.3 x 10^-3 | -0.02 | 0.50 |

**Bacteria**

| **Relationship** | **β** | **R²_adj** | **λ** |
| --- | --- | --- | --- |
| **vs. Genome Size** | -7.2 x 10^-5** | 0.03 | 0.91 |
| **vs. Gene Content** | -7.2 x 10^-5** | 0.02 | 0.91 |
| **vs. Coding** | -7.3 x 10^-5 | 0.02 | 0.91 |
| **vs. Gene / Genome** | 1.6 x 10^-3** | 0.02 | 0.91 |
| **vs. Coding / Gene** | -0.02** | 0.02 | 0.92 |
| **vs. Coding / Genome** | 1.5 x 10^-3** | 0.01 | 0.91 |

**Archaea**

| **Relationship** | **β** | **R²_adj** | **λ** |
| --- | --- | --- | --- |
| **vs. Genome Size** | -8.3 x 10^-5 | -3.0 x 10^-4 | 0.97 |
| **vs. Gene Content** | -6.4 x 10^-5 | -4.0 x 10^-3 | 0.97 |
| **vs. Coding** | -6.7 x 10^-5 | -3.8 x 10^-3 | 0.97 |
| **vs. Gene / Genome** | 0.01*** | 0.07 | 0.97 |
| **vs. Coding / Gene** | -0.13 | 0.13 | 0.96 |
| **vs. Coding / Genome** | 4.9 x 10^-3*** | 0.06 | 0.97 |

**Part B: ASR* models**

**Mammals**

| **Relationship** | **β** | **R²_adj** | **λ** |
| --- | --- | --- | --- |
| **vs. Genome Size** | 3.7 x 10^-4** | 0.03 | 0.25 |
| **vs. Gene Content** | 1.9 x 10^-3*** | 0.32 | 0.25 |
| **vs. Coding** | 0.12** | 0.06 | 0.25 |
| **vs. Gene / Genome** | 4.44*** | 0.18 | 0.25 |
| **vs. Coding / Gene** | -82.74*** | 0.27 | 0.18 |
| **vs. Coding / Genome** | -37.77 | -3.0 x 10^-3 | 0.50 |

**Birds**

| **Relationship** | **β** | **R²_adj** | **λ** |
| --- | --- | --- | --- |
| **vs. Genome Size** | 6.68 x 10^-4** | 0.06 | 0.07 |
| **vs. Gene Content** | 2.2 x 10^-3*** | 0.29 | 0.25 |
| **vs. Coding** | 0.08** | 0.13 | 0.10 |
| **vs. Gene / Genome** | 1.79** | 0.11 | 0.08 |
| **vs. Coding / Gene** | -35.67*** | 0.25 | 0.25 |
| **vs. Coding / Genome** | -14.96 | -0.01 | 0.11 |

**Fish**

| **Relationship** | **β** | **R²_adj** | **λ** |
| --- | --- | --- | --- |
| **vs. Genome Size** | 1.42 x 10^-4** | 0.03 | 0.70 |
| **vs. Gene Content** | 1.48 x 10^-4 | 0.01 | 0.69 |
| **vs. Coding** | 4.2 x 10^-4 | -0.01 | 0.69 |
| **vs. Gene / Genome** | -1.37*** | 0.12 | 0.66 |
| **vs. Coding / Gene** | -2.23** | 0.03 | 0.68 |
| **vs. Coding / Genome** | -5.56*** | 0.09 | 0.67 |

**Arthropods**

| **Relationship** | **β** | **R²_adj** | **λ** |
| --- | --- | --- | --- |
| **vs. Genome Size** | 7.29 x 10^-5** | 0.02 | 0.74 |
| **vs. Gene Content** | 3.23 x 10^-4** | 0.07 | 0.72 |
| **vs. Coding** | 0.02** | 0.05 | 0.75 |
| **vs. Gene / Genome** | -0.36 | 4.4 x 10^-3 | 0.76 |
| **vs. Coding / Gene** | -3.19*** | 0.23 | 0.59 |
| **vs. Coding / Genome** | -5.29*** | 0.26 | 0.56 |

**Plants**

| **Relationship** | **β** | **R²_adj** | **λ** |
| --- | --- | --- | --- |
| **vs. Genome Size** | 8.23 x 10^-5*** | 0.15 | 0.88 |
| **vs. Gene Content** | 1.15 x 10^-3*** | 0.18 | 0.84 |
| **vs. Coding** | 3.8 x 10^-3** | 0.07 | 0.87 |
| **vs. Gene / Genome** | -0.85*** | 0.15 | 0.91 |
| **vs. Coding / Gene** | -1.16*** | 0.10 | 0.80 |
| **vs. Coding / Genome** | -3.24*** | 0.23 | 0.87 |

**Fungi**

| **Relationship** | **β** | **R²_adj** | **λ** |
| --- | --- | --- | --- |
| **vs. Genome Size** | 2.4 x 10^-4 | -0.01 | 0.25 |
| **vs. Gene Content** | 3.8 x 10^-3** | 0.11 | 0.50 |
| **vs. Coding** | 1.7 x 10^-3 | -8.5 x 10^-4 | 0.50 |
| **vs. Gene / Genome** | 0.14** | 0.06 | 0.20 |
| **vs. Coding / Gene** | -0.35*** | 0.26 | 0.50 |
| **vs. Coding / Genome** | -0.03 | -0.01 | 0.25 |

**Unicellular Eukaryotes**

| **Relationship** | **β** | **R²_adj** | **λ** |
| --- | --- | --- | --- |
| **vs. Genome Size** | -9.2 x 10^-6 | -0.02 | 0.25 |
| **vs. Gene Content** | -2.40 x 10^-5 | -0.02 | 0.25 |
| **vs. Coding** | -1.2 x 10^-5 | -0.03 | 0.25 |
| **vs. Gene / Genome** | -1.4 x 10^-4 | -0.03 | 0.25 |
| **vs. Coding / Gene** | 0.01 | -4.6 x 10^-3 | 0.25 |
| **vs. Coding / Genome** | 1.6 x 10^-3 | -0.03 | 0.25 |

**Bacteria**

| **Relationship** | **β** | **R²_adj** | **λ** |
| --- | --- | --- | --- |
| **vs. Genome Size** | -7.30 x 10^-5** | 0.03 | 0.91 |
| **vs. Gene Content** | -7.16 x 10^-5** | 0.02 | 0.91 |
| **vs. Coding** | -7.28 x 10^-5** | 0.02 | 0.91 |
| **vs. Gene / Genome** | 1.6 x 10^-3** | 0.02 | 0.91 |
| **vs. Coding / Gene** | -0.02** | 0.02 | 0.92 |
| **vs. Coding / Genome** | 1.5 x 10^-3** | 0.02 | 0.91 |

**Archaea**

| **Relationship** | **β** | **R²_adj** | **λ** |
| --- | --- | --- | --- |
| **vs. Genome Size** | -8.31 x 10^-5 | -3.0 x 10^-4 | 0.97 |
| **vs. Gene Content** | -6.38 x 10^-5 | -4.0 x 10^-3 | 0.97 |
| **vs. Coding** | -6.67 x 10^-5 | -3.8 x 10^-3 | 0.97 |
| **vs. Gene / Genome** | 0.01*** | 0.07 | 0.97 |
| **vs. Coding / Gene** | -0.13*** | 0.13 | 0.96 |
| **vs. Coding / Genome** | 4.8 x 10^-3*** | 0.07 | 0.97 |
