# Supplementary material for: Alternative splicing across the tree of life
Source: eLife. 2025 Oct 17;13:RP94802. doi: 10.7554/eLife.94802 (PMC12534046; doi:10.7554/eLife.94802)
Supplement: Supplementary file 4. [file elife-94802-supp4.docx]

Coefficient of variation (CV̂) for each genomic variable and taxonomic group. It is computed as CV̂ = s / x̄, where s is the standard deviation and x̄ the mean. Columns correspond to Genome size, Gene content, Coding content, Gene/Genome (%), Coding/Gene (%), Coding/Genome (%), the Alternative Splicing Ratio (ASR), and its normalized ratio ASR*.

|  | CV̂ Genome | CV̂ Gene | CV̂ Coding | CV̂ Gene / Genome | CV̂ Coding / Gene | CV̂ Coding / Genome | CV̂ ASR | CV̂ ASR* |
| --- | --- | --- | --- | --- | --- | --- | --- | --- |
| Mammals | 0.13 | 0.16 | 0.04 | 0.14 | 0.14 | 0.11 | 0.30 | 0.21 |
| Birds | 0.10 | 0.12 | 0.05 | 0.12 | 0.09 | 0.08 | 0.24 | 0.12 |
| Fish | 0.57 | 0.47 | 0.23 | 0.12 | 0.29 | 0.33 | 0.18 | 0.14 |
| Arthropods | 1.87 | 1.28 | 0.19 | 0.23 | 0.63 | 0.63 | 0.25 | 0.17 |
| Plants | 1.11 | 0.58 | 0.38 | 0.45 | 0.27 | 0.54 | 0.13 | 0.11 |
| Fungi | 0.75 | 0.51 | 0.43 | 0.22 | 0.13 | 0.28 | 0.05 | 0.05 |
| Uni. Euk. | 1.02 | 0.79 | 0.72 | 0.27 | 0.14 | 0.28 | 0.00 | 0.00 |
| Bacteria | 0.53 | 0.53 | 0.54 | 0.06 | 0.01 | 0.06 | 0.00 | 0.00 |
| Archaea | 0.38 | 0.34 | 0.34 | 0.06 | 0.00 | 0.06 | 0.00 | 0.00 |
