# Supplementary material for: Alternative splicing across the tree of life
Source: eLife. 2025 Oct 17;13:RP94802. doi: 10.7554/eLife.94802 (PMC12534046; doi:10.7554/eLife.94802)
Supplement: Supplementary file 5. [file elife-94802-supp5.docx]

Relative variability among genomic features, computed as ratios of coefficients of variation (CV̂): CV̂ₓ / CV̂ᵧ, where CV̂ = s / x̄ (standard deviation divided by the sample mean). Values are computed separately for each taxonomic group. The first table compares variability among genome composition variables; the second and third compare these variables with ASR and normalized ASR (ASR*), respectively.

|  | CV̂_Gene / CV̂_Genome | CV̂_Coding / CV̂_Gene | CV̂_Coding / CV̂_Genome | CV̂_Gene/Genome / CV̂_Genome | CV̂_Coding/Gene / CV̂_Gene | CV̂_Coding/Genome / CV̂_Genome |
| --- | --- | --- | --- | --- | --- | --- |
| Mammals | 1.22 | 0.25 | 0.30 | 1.04 | 0.85 | 0.83 |
| Birds | 1.19 | 0.40 | 0.48 | 1.11 | 0.77 | 0.80 |
| Fish | 0.83 | 0.48 | 0.40 | 0.21 | 0.61 | 0.58 |
| Arthropods | 0.69 | 0.15 | 0.10 | 0.12 | 0.49 | 0.34 |
| Plants | 0.52 | 0.65 | 0.34 | 0.40 | 0.47 | 0.49 |
| Fungi | 0.68 | 0.84 | 0.57 | 0.29 | 0.26 | 0.37 |
| Uni. Euk. | 0.77 | 0.91 | 0.70 | 0.26 | 0.17 | 0.27 |
| Bacteria | 1.00 | 1.01 | 1.01 | 0.11 | 0.01 | 0.11 |
| Archaea | 0.89 | 1.00 | 0.90 | 0.16 | 0.01 | 0.16 |

|  | CV̂_ASR / CV̂_Genome | CV̂_ASR / CV̂_Gene | CV̂_ASR / CV̂_Coding | CV̂_ASR / CV̂_Gene/Genome | CV̂_ASR / CV̂_Coding/Gene | CV̂_ASR / CV̂_Coding/Genome |
| --- | --- | --- | --- | --- | --- | --- |
| Mammals | 2.22 | 1.82 | 7.34 | 2.13 | 2.15 | 2.67 |
| Birds | 2.30 | 1.94 | 4.79 | 2.06 | 2.50 | 2.89 |
| Fish | 0.32 | 0.38 | 0.79 | 1.50 | 0.62 | 0.55 |
| Arthropods | 0.13 | 0.20 | 1.32 | 1.08 | 0.40 | 0.40 |
| Plants | 0.12 | 0.22 | 0.34 | 0.29 | 0.47 | 0.24 |
| Fungi | 0.07 | 0.10 | 0.11 | 0.23 | 0.37 | 0.18 |
| Uni. Euk. | 0.00 | 0.00 | 0.00 | 0.01 | 0.02 | 0.01 |
| Bacteria | 0.00 | 0.00 | 0.00 | 0.01 | 0.14 | 0.01 |
| Archaea | 0.00 | 0.01 | 0.01 | 0.03 | 0.77 | 0.03 |

|  | CV̂_ASR* / CV̂_Genome | CV̂_ASR* / CV̂_Gene | CV̂_ASR* / CV̂_Coding | CV̂_ASR* / CV̂_Gene/Genome | CV̂_ASR* / CV̂_Coding/Gene | CV̂_ASR* / CV̂_Coding/Genome |
| --- | --- | --- | --- | --- | --- | --- |
| Mammals | 1.57 | 1.28 | 5.19 | 1.51 | 1.52 | 1.89 |
| Birds | 1.21 | 1.02 | 2.51 | 1.08 | 1.31 | 1.51 |
| Fish | 0.25 | 0.30 | 0.63 | 1.19 | 0.50 | 0.44 |
| Arthropods | 0.09 | 0.13 | 0.89 | 0.73 | 0.27 | 0.27 |
| Plants | 0.10 | 0.19 | 0.30 | 0.25 | 0.41 | 0.21 |
| Fungi | 0.07 | 0.10 | 0.11 | 0.23 | 0.37 | 0.18 |
| Uni. Euk. | 0.00 | 0.00 | 0.00 | 0.01 | 0.02 | 0.01 |
| Bacteria | 0.00 | 0.00 | 0.00 | 0.01 | 0.14 | 0.01 |
| Archaea | 0.00 | 0.01 | 0.01 | 0.03 | 0.77 | 0.03 |
